# Use of Chemical Nano-Selenium as an Antibacterial and Antifungal Agent in Quail Diets and Its Effect on Growth, Carcasses, Antioxidant, Immunity and Caecal Microbes

**DOI:** 10.3390/ani11113027

**Published:** 2021-10-21

**Authors:** Mahmoud Alagawany, Shaza Y. A. Qattan, Youssef A. Attia, Mohamed T. El-Saadony, Shaaban S. Elnesr, Mohamed A. Mahmoud, Mahmoud Madkour, Mohamed E. Abd El-Hack, Fayiz M. Reda

**Affiliations:** 1 Poultry Department, Faculty of Agriculture, Zagazig University, Zagazig 44511, Egypt; dr.mohamed.e.abdalhaq@gmail.com (M.E.A.E.-H.); fayizreda@yahoo.com (F.M.R.); 2 Biological Sciences Department, Microbiology, Faculty of Science, King Abdulaziz University, Jeddah 80203, Saudi Arabia; syqattan@kau.edu.sa; 3 Agriculture Department, Faculty of Environmental Sciences, King Abdulaziz University, Jeddah 21589, Saudi Arabia; 4 Department of Agricultural Microbiology, Faculty of Agriculture, Zagazig University, Zagazig 44511, Egypt; m.elsadony@zu.edu.eg; 5 Poultry Production Department, Faculty of Agriculture, Fayoum University, Fayoum 63514, Egypt; ssn00@fayoum.edu.eg; 6 Department of Physiology, Faculty of Veterinary Medicine, New Valley University, New Valley 72511, Egypt; barhoma56@gmail.com; 7 Animal Production Department, National Research Centre, Dokki, Giza 12622, Egypt; mahmoud.madkour9@gmail.com

**Keywords:** nano particles, selenium, performance, blood, pathogens, quails

## Abstract

**Simple Summary:**

The chemical Nano-Selenium (Che-SeNPs) is a good example of applied nanotechnology used in the area of nutritional supplements due to its advantages and properties. From our results, dietary supplementation with Che-SeNPs could improve the performance of growing quails; the best level was 0.4 g Che-SeNPs/kg feed. Thus, this study supports the application of Che-SeNPs in quail diets in an effort to improve the productive and physiological performance. The results revealed that Che-SeNPs boosts the growth, blood biochemistry, antioxidant indices, immunity, and bacterial environment of the intestine of quail.

**Abstract:**

Nano-minerals are used to enhance mineral bioavailability, which helps improve animal growth and health. The use of chemical nano-selenium (Che-SeNPs) has lately attracted great scientific interest, mainly due to its potential benefits for poultry. The current study was conducted to investigate the impact of the dietary supplementation of Che-SeNPs on the growth performance, carcass traits, blood constituents, antioxidant status, immunity, and gut microbiota of Japanese quails. A total of one week-old 180 Japanese quails were randomly distributed into four equal groups, and each group consisted of 45 unsexed birds with five replications (nine birds each). The first group was fed a basal diet without supplementation (0 g/kg Che-SeNPs), and the second, third, and fourth groups were fed diets containing 0.2, 0.4, and 0.6 g/kg Che-SeNPs, respectively. The results showed that the dietary supplementation of Che-SeNPs significantly (*p* < 0.0001) increased body weight, body weight gain, and feed conversion ratio, but decreased feed intake (*p* < 0.0001) compared to the control group. The highest values of growth performance were recorded in the group fed 0.4 g Che-SeNPs g/kg feed. Che-SeNPs levels did not affect the carcass traits, relative organs (except liver), or blood hematology (except platelet count and hemoglobin level) of quails. Plasma total protein, albumin, aspartate amino transferase (AST), and urea values were not affected by dietary Che-SeNPs, but alanine aminotransferase and lactate dehydrogenase values declined. Globulin and creatinine values were linearly increased with the inclusion of Che-SeNPs (0.4 and 0.6 g/kg) in quail diets compared to the control. The supplementation of Che-SeNPs in quail diets significantly improved (*p* < 0.05) the plasma lipid profile and activities of antioxidant enzymes compared to the control group. Immunoglobulin G values of Che-SeNPs (0.4 and 0.6 g/kg) were higher (*p* < 0.05) than those in the control group. The groups fed diets supplemented with Che-SeNPs showed lower (*p* < 0.0001) total bacterial count, total yeast and molds count, *Coliform*, *Escherichia coli*, *Enterococcus* spp., and *Salmonella* spp. colonization, and higher (*p* = 0.0003 and 0.0048) lactic acid bacteria counts than those in the control group. In conclusion, Che-SeNPs supplemented up to 0.4 g/kg can improve the performance, lipid profile, antioxidant indices, and immunity, as well as decrease intestinal pathogens in quails during the fattening period (1–5 weeks of age).

## 1. Introduction

Selenium (Se) is one of the elements that can be used in diets as the chemical nano-selenium (Che-SeNPs). Se is required for the maintenance of physiological functions, growth, and health of birds. It also plays a crucial role in nutritional value and feed metabolism, leading to considerable growth [1]. Che-SeNPs has attracted more attention because of its strong adsorbing ability, high catalytic efficiency, high surface activity, and low toxicity compared to that of other chemical Se forms [2]. The high absorption of Che-SeNPs from the intestinal lumen into the body was observed. Shirsat et al. [3] highlighted that Che-SeNPs has antioxidant, anticancer, antibacterial, and antiprotozoal properties. El-Deep et al. [4] stated that dietary Che-SeNPs supplementation enhanced growth performance by improving immune or antioxidative properties in broiler chicks. Additionally, Ahmadi et al. [5] revealed that the dietary supplementation of Che-SeNPs improved growth performance and immune function without the deleterious effects on the internal organs of broiler chickens.

Previous investigations exhibited that Che-SeNPs augmented body weight gain and improved antioxidant functions of Arbor Acres broilers [3,6]. Se nanoparticles have also been utilized in food preservation methods such as packing food items and antiseptic coating over food materials. Studies have been conducted to highlight the disinfectant properties of Se nanoparticles against *Pseudomonas aeruginosa*, and *Proteus mirabilis* [7]. On the other hand, natural agents and trace elements including nanoparticles as feed additives may affect the diversity of gut microbiota and health [8]. Se is one of the important elements that can help microbiota to complete its action within the gut [9]. In this concern, the caecal counts of *Salmonella* and *E. coli* of quails were decreased in birds fed diets containing nano-curcumin when compared to the control diets [8].

Selenium can be considered an essential trace element and micronutrient for living creatures at low concentrations, but it becomes toxic and harmful at higher dose [2]. The extensive use of nano Se in nanotechnologies and medicine has increased the risk of their contamination in the environment, which could harm living species; however, it is useful to understand the assessment of Se-NPs toxicity to the biological ecosystem [10]. Nano-Se has lower toxicity than selenomethionine and is now the least toxic of all supplments of Se. Nano-Se has a threefold lower toxicity than organic Se and a sevenfold lower toxicity than inorganic Se [10].

The positive impacts of nanotechnology involving Se are well-known in many pathological conditions [11]. However, the inclusion of Che-SeNPs in quail diet during the growth period remains limited. It is hypothesized that the dietary addition of Che-SeNPs is expected to exert beneficial effects on growing quails. Therefore, the purpose of this study was to evaluate the antibacterial and antifungal activities of Che-SeNPs, and its beneficial effects on the growth, feed utilization, carcass traits, hematology, blood constituents, and cecal microbiota of growing quails.

## 2. Materials and Methods

### 2.1. Source of Selenium Nanoparticles

The study was carried out at Zagazig University, Zagazig, Egypt in conjunction with King Abdulaziz University, Jeddah, Saudi Arabia under protocol no: (FP-73-43). In this study, Che-SeNPs were prepared using wet chemicals. Sodium selenite (Na_2_SeO_3_) was used for producing Se nanoparticles with ascorbic acid (C_6_H_8_O_6_) as a reducing agent. A stock of aqueous solution of 100 mM Na_2_SeO_3_ and 50 mM C_6_H_8_O_6_ was prepared in a 1: 4 ratio. The solution was kept under a magnetic stirring condition at different rpm and ambient temperature for 30 min. The mixtures were allowed to react with each other in the concentrated form until the mixture changed from colorless to red. Next, the solution was centrifuged at 3000 rpm, pellets were collected, and Che-SeNPs was obtained [12,13]. Chemically synthesized nano-selenium was determined via UV–Vis spectroscopy using an automated spectrometer (Spectro UV–Vis double beam UVD 3500). The morphology and element percentage of selenium nanoparticles were measured using transmission electron microscopy and an energy dispersive X-ray analytical instrument. Fourier transform infrared spectroscopy (JASCO) was used to determine the properties of produced selenium nanoparticles including size, shape, charge, and stability. Characterization of Che-SeNPs; maximum UV absorbance at 300 nm, spherical shape by TEM, size (75.68 nm) and charge (−23.26 mV) by zeta seizer, and zeta potential, respectively.

### 2.2. Antibacterial Activity of Che-SeNPs

*Listeria monocytogenes* ATCC 15313, *Staphylococcus aureus* MTCC 1809, *Bacillus cereus* ATCC 11778, *E. coli* ATCC 25922, *P. aeruginosa* ATCC 27853, and *Salmonella enterica* MTCC 1253 were used in this study. The antibacterial activity of Che-SeNPs against animal and human pathogenic Gram-negative bacteria, *P. aeruginosa* ATCC 27853, *E. coli* ATCC 25922, and *S. enterica* MTCC 1253, and Gram-positive bacteria, *L. monocytogenes* ATCC 15313, *B. cereus* ATCC 11778, and *S. aureus* MTTC 1809, were estimated using the disc diffusion assay method. Mueller–Hinton agar medium consisting of peptone, beef extract, yeast extract, NaCl, and agar with 5, 3, 5, 5, and 20 g, respectively in 1 L of distilled water was prepared in slant to preserve all bacterial strains. Mueller–Hinton broth was used to activate bacterial cells; one hundred microliters of each bacterium (1 × 10^9^ CFU/mL) were spread with sterile swabs in Mueller–Hinton agar plates. Freshly prepared selenium nanoparticles with different concentrations (50, 100, 200, 400, and 800 μg/mL) were loaded on paper discs (disc diameter was about 6 mm) and then were placed on the Muller–Hinton agar plates. Sodium selenite (50 μg/mL) and sterilized deionized water were loaded on paper discs and used as a positive and negative control, respectively. Mueller-Hinton agar plates were incubated for 24 h at 37 °C. After incubation, the obtained zones of inhibition surrounded the Che-SeNPs discs were measured and recorded as the mean ± standard deviation if they were greater than 6 mm. The minimum inhibitory concentration (MIC) of the Che-SeNPs was calculated based on a broth micro dilution method. Briefly, six pathogenic bacteria were cultured overnight at 37 °C in Mueller–Hinton broth and were adjusted to a final density of 10^9^ CFU/mL by 0.5 McFarland standards. The Che-SeNPs (1 mg/mL) were homogenized with sterilized deionized water and dilutions of 50, 100, 200, 400, and 800 μg/mL were made. Next, 10 μL of different concentrations of Che-SeNPs was mixed in sterile test tubes contain 10 μL of bacterial inoculum and 90 μL of Mueller–Hinton broth. The test tubes were incubated for a day at 37 °C. The lower concentration of Che-SeNPs which inhibited bacterial strains growth or turbidity was considered the MIC. The lower concentration of Che-SeNPs which totally killed bacterial strains was defined as the minimum bactericidal concentration (MBC). The experiments were carried out in triplicate [8].

### 2.3. Antifungal Activity of Che-SeNPs

The antifungal activity of the Che-SeNPs was tested against animals and human pathogenic *Candida* strains. *Candida albicans* ATCC 4862, *C. glabrata*ATCC64677, *C. parapsilosis* ATCC 22019, and *C. guilliermondii* ATCC 6260 were used in this study. The antifungal activity of Che-SeNPs against these four strains was evaluated via the disc diffusion method [14] using sterile cotton swab lawn cultures of selected fungi that were prepared on Sabouraud Dextrose agar (SDA) plates. Che-SeNPs was loaded on paper discs (disc diameter was about 6 mm) and then was placed in SDA surface. Selenium selenite and sterilized deionized water were used as the positive and negative controls, respectively. The plates were then incubated for 36h at 30 °C. The Che-SeNPs were tested for MIC using the broth dilution method [14]. Sabouraud broth was used as diluents for fungal species. About 10^6^ CFU/mL cells could be inoculated. The Che-SeNPs levels (50 to 800 μg/mL) were prepared in sterilized deionized water and homogenized. Next, 10 μL of different concentrations of Che-SeNPs were mixed in sterile test tubes containing 10 μL of *Candida* inoculum and 90 μL of Sabouraud broth. The test tubes were incubated at 30 °C for 36 h. The obtained turbidity was estimated at 600 nm to determine the MIC values. The minimum concentration of Che-SeNPs that reduced fungi growth by 90% was considered the minimum inhibitory concentration (MIC). The concentration of Che-SeNPs at which complete fungal growth was not observed was defined as the minimum fungicidal concentration (MFC). The experiments were replicated in triplicate.

### 2.4. Animals, Design, and Diets 

A total of 180 one-week-old Japanese quails with an average weight of 27.17 ± 0.075 g were used. Quail chicks were randomly allocated into four groups, and each group consisted of 45 unsexed birds with five replications (nine birds each). Quails were kept in conventional cages (90 × 40× 40 cm), and feed and water were open during the study (4 weeks). The treatments were as follows: the first group was fed the basal ration which containing 150 mg of Se as Se selenite, whereas the second, third, and fourth groups were fed diets supplemented with 0.2, 0.4, and 0.6 g/kg of Che-SeNP, respectively. The Che-SeNP was added at the top of the basal diet at the highest level and then diluted with the unsupplemented basal diet to achieve the desire concentration. The basal diet (Table 1) was formulated to meet the birds’ requirements according to NRC [15]. The Ethics statement for Animal care and maintenance were in accordance with the guidelines of the Egyptian Research Ethics Committee and the guidelines for the Care and Use of Laboratory Animals by Zagazig University (ZU-IACUC/2/F/56/2021).

### 2.5. Growth Performance and Carcass Measurements

All growth parameters and feed utilization were measured at 1, 3, and 5 weeks of age. For carcass examinations, at 5weeks old, 20 birds (5 per treatment) were randomly selected, weighed, and euthanized. All edible parts were weighed and expressed as a percent of the live body weight before slaughter.

### 2.6. Microbiological Analysis

Ten grams of quail cecum samples (five samples per each treatment) were homogenized and transferred to a 250 mL conical flask containing 90 mL of sterile physiological saline solution consisting of 0.1% peptone and 0.85%NaCl; the mixture was well-mixed to obtain a 10^−1^ dilution. Serial dilutions from the previous dilution (10^−1^) were prepared to obtain up to 10^−6^ dilution. The total bacterial count was counted using plate count agar medium at 30 °C for 24 h, and the total count of *Enterococcus* spp., was counted using Chromocult enterococci agar medium [16,17]. Total coliforms were enumerated by using MacConkey agar medium. Biochemical methods such as indole test, citrate reactions, methyl red, and Voges–Proskauer were used to identify *Escherichia coli*. DeManRogosa Sharpe agar was used to enumerate the lactic acid bacteria. *Salmonella Shigella* agar (SSA) media (Oxide CM 99) was used to count the *Salmonella* spp. The appearance of black colonies on SSA indicated the presence of *Salmonella* spp. SSA plates were incubated at 37 °C for 1 day. Sabouraud Dextrose agar (SDA) was used to count the molds and yeasts. SDA plates were incubated at 25 °C for 3–7 days. All the obtained microbiological results were then converted to logarithmic colony-forming units per gram (CFU/g) [8,18,19].

### 2.7. Blood Chemistry 

After euthanization, blood samples were randomly collected from five quails per treatment into heparinized tubes. Hematological parameters were measured. Regarding biochemical parameters, we used a centrifuge (Janetzki, T32c, 5000 rpm, Germany) at 2146.56× *g* for 15 min to separate the plasma. The biochemical blood parameters were determined using commercial kits from Biodiagnostic Company (Giza, Egypt).

### 2.8. Statistics

The statistical analyses were carried out using SAS. The data of growth rate, feed efficiency, carcass parameters, hematology, blood chemistry, and microbiology were analyzed with a one-way analysis of variance using a normal distribution and the replicate as the experimental unit. Orthogonal polynomial contrasts were used to test the significance (linear and quadratic) of the gradual levels of dietary Che-SeNPs using the post-hoc Tukey’s test (*p* < 0.05).

## 3. Results

### 3.1. Antibacterial Activity of Che-SeNPs

Three animal and human pathogenic Gram-negative bacteria (*E. coli, P. aeruginosa*, and *S. enterica*) and three Gram-positive bacteria (*L. monocytogenes*, *B. cereus*, and *S. aureus*) were selected to test Che-SeNPs antibacterial activity (Table 2). The maximum zones of inhibitions were observed in the three Gram-positive bacterial strains *L. monocytogenes* ATCC 15313, *B. cereus* ATCC 11778, and *S. aureus* MTTC 1809. The antibacterial activity of Che-SeNPs increased with increasing concentrations of Che-SeNPs. The effect of Che-SeNPs was superior to that of sodium selenite as an antimicrobial agent against tested pathogenic microorganisms; moreover, the deionized water did not show any antimicrobial activity. The highest MIC of Che-SeNPs against *E. coli* ATCC 25922, *P. aeruginosa* ATCC 27853, and *S. enterica* MTCC 1253 was 45, 40, and 50μg/mL, respectively, whereas, the lowest MIC was 30, 35, and 25 μg/mL against *L. monocytogenes* ATCC 15313, *S. aureus* MTTC 1809, and *B. cereus* ATCC 11778, respectively (Table 2).

### 3.2. Antifungal Activity of Che-SeNPs

Che-SeNPs showed acceptable antifungal activity, ranging from 50 μg/mL to 800 μg/mL, against all the tested fungal strains. *C. albicans* ATCC 4862 was the most sensitive strain to Che-SeNP when compared to other tested strains. The MICs for *C. albicans* ATCC 4862, *C. glabrata* ATCC64677, *C. parapsilosis* ATCC 22019, and *C. guilliermondii* ATCC 6260 were 70, 90, 80, and 100 μg/mL, respectively (Table 3). 

### 3.3. Growth Performance 

The effects of dietary Che-SeNPs supplementation on the growth performance of Japanese quails are presented in Table 4. From the results, quails fed diets containing Che-SeNPs had significantly higher body weight (BW) (linear, *p* < 0.0001 and quadratic, *p* = 0.0004) and body weight gain (BWG) (linear, *p* < 0.0001 and quadratic, *p* = 0.0005) during the whole experimental period. The group fed diets containing Che-SeNPs (0.4 g/kg diet) had the highest BW and BWG. Feed intake was decreased (linear and quadratic, *p* < 0.001) in the Che-SeNPs groups compared with that in the control group. The feed conversion ratio was linearly and quadratically improved with the addition of Che-SeNPs in quail diets during all periods. Generally, the best growth performance parameters were recorded in the group fed 0.4 g Che-SeNPs/kg feed.

### 3.4. Carcass Traits 

As indicated in Table 5, dietary Che-SeNPs levels did not affect carcass traits and relative organs (except liver) of Japanese quails. Supplementation of Che-SeNPs significantly increased the relative weight of the liver (linear and quadratic, *p* < 0.05) compared to the control group.

### 3.5. Blood Hematology

The effects of the addition of Che-SeNPs on the blood hematology of growing quails are presented in Table 6. Dietary Che-SeNPs levels did not affect (*p* > 0.05) white blood cells (WBCs), lymphocytes, mid-range, granulocytes, red blood cells (RBCs), hematocrit, and mean corpuscular volume of the growing quails. Platelet count and hemoglobin (Hb) levels were increased (linear, *p* < 0.05) by the addition of Che-SeNPs at 0.4 and 0.6 g/kg. The mean corpuscular volume value was augmented (linear, *p* < 0.05) by the addition of Che-SeNPs level compared to the control group (without Che-SeNPs). The supplementation of dietary Che-SeNPs at levels of 0.6 g/kg decreased values of red blood cell distribution width linearly (*p* = 0.0091). In contrast, the dietary levels of 0.4 and 0.6 g/kg declined the values of red blood cell distribution volume linearly (*p* = 0.0019) compared to the control group.

### 3.6. Blood Constituents

Liver and kidney function data are presented in Table 7. The total protein and albumin were not affected (*p* > 0.05) by dietary Che-SeNPs. The globulin value was lowest (linear, *p* < 0.05) in the Che-SeNPs levels of 0.4 and 0.6 g/kg compared with the Che-SeNPs levels of 0.2 g/kg and the control group. The quails fed diets containing Che-SeNPs had higher alanine aminotransferase (ALT) and lactate dehydrogenase (*p* < 0.05) than those in the control group. Dietary Che-SeNPs had no significant effect on AST and urea values. The inclusion of Che-SeNPs (0.4 and 0.6 g/kg) in quail diets increased the creatinine value (linear, *p* < 0.05) compared with that in the control and 0.2 g/kg Che-SeNPs groups.

The effects of Che-SeNPs inclusion in diets on the lipid profile of quails are presented in Table 8. Total cholesterol, triglyceride, and very-low-density lipoprotein were significantly decreased (*p* < 0.05) in Che-SeNPs-treated groups compared to those in control. The dietary supplementation of Che-SeNPs (0.2 and 0.4 g/kg) quadratically increased high-density lipoprotein (HDL) (*p* = 0.0019).

### 3.7. Antioxidant Indices

The results of the antioxidant indices in the serum are given in Table 8. The activities of superoxide dismutase (SOD) and glutathione peroxidase, and the levels of reduced glutathione (GSH) were significantly increased (linear and quadratic, *p* < 0.05) by the dietary supplementation of Che-SeNPs compared with those in control. Dietary Che-SeNPs levels decreased malondialdehyde (MDA) levels linearly (*p* < 0.0001) compared to the control group. The values of immunoglobulin G (IgG) of Che-SeNPs-treated groups were higher (linear and quadratic, *p* < 0.05) than those in the control group. IgM and IgA values of Che-SeNPs (0.4 and 0.6 g/kg) were higher (*p* < 0.05) than those in the control group. The quails fed a diet supplemented with Che-SeNPs showed higher plasma selenium concentrations when compared to those fed the control diet (linear, *p* = 0.0001).

### 3.8. Microbiological Analysis

The different Che-SeNPs levels significantly affected the cecal microbiota of growing Japanese quails (Table 9). The groups fed a diet supplemented with Che-SeNPs showed lower total bacterial count, total yeast and molds count, *Coliform*, *E. coli*, *Enterococcus* ssp., and *Salmonella* spp. colonization than those in the control group (linear and quadratic, *p* < 0.0001). However, the dietary supplementation of Che-SeNPs levels increased the lactic acid bacteria count (linear and quadratic, *p* < 0.05) compared to the control group.

## 4. Discussion 

Antimicrobial agents are critical in the pharmaceutical and textile industries, water purification, and food packaging. One notable disadvantage of organically synthesized compounds is toxicity in the body; therefore, the trend is to use inorganic nanoparticles such as Che-SeNPs with antimicrobial activity [20]. These Che-SeNPs have an inhibitory effect on many microorganisms. Currently, antimicrobial drugs are becoming less effective for many diseases globally because of the drug resistance capability of microbes. Microorganisms use their biofilm to resist antimicrobial drugs, and the membranes are the primary source of food contamination. Che-SeNPs have been used to control the growth and formation of biofilms of food spoilage bacteria, including *B. cereus*, *Enterococcus faecalis*, *S. aureus*, *E. coli* O157:H7, *S. typhimurium*, and *S. enterica* [21]. The development more effective antibacterial agents is vital for a wide range of applications in various diseases for better public health. However, the emergence of multiple antibiotic-resistant bacteria presents a public health threat. Many developed antimicrobial drugs have limited effective applications due to chemical imbalances, low biocompatibility, and poor long-term antibacterial efficiency. Che-SeNPs conjugated with quercetin and acetylcholine have shown a tremendous antimicrobial effect on the pathogen [22]. Probiotics are microorganisms that can improve intestinal microbial balance and benefit poultry health after consumption in adequate amounts. *Lactobacillus plantarum* and *L. johnsonii* cells are resistant against selenium dioxide, and their cell-free extracts were tested against *C*. *albicans* ATCC 14053 [7]. Selenium particles extracted from cultures of *S. carnosus* stabilized by their natural protein coating, for instance, show considerable activity against the nematode *Steinernema feltiae*, *Saccharomyces cerevisiae,* and *E. coli*. Natural SeNPs were found to be more active than mechanically generated selenium particles and can be applied as antimicrobial materials in medicine and agriculture [23]. Antimicrobial tests show SeNPs activity against *S. epidermidis*, but not against *E*. *coli* in a low Se concentration of 2 ppm. *S. aureus* is an important bacterium commonly found in numerous infections. *S. aureus* infections were difficult to treat due to their biofilm formation and defined antibiotic resistance. SeNPs were used effectively in the prevention and treatment of disease caused by *S. aureus* [8].

The antifungal activity of SeNPs was evaluated against *C. albicans* ATCC 4862, *C. glabrata* ATCC64677, *C. parapsilosis* ATCC 22019, and *C. guilliermondii* ATCC 6260 using the disk diffusion method [14] (Table 2). The common antifungal agents are enormously irritant and lethal, and it is necessary to formulate newer types of safe and cost-effective fungicidals. Accordingly, the present study illustrates that selenium nanoparticles have good antifungal activity against all pathogenic animals and human *Candida* species. Selenium nanoparticles showed better activity against *C. albicans* ATCC 4862 compared to other *Candida* species used in this study. In addition, it was proved that SeNPs ranging in size from 100 to 550 nm, with an average size of 245 nm, have low toxicity and high biological activities [24]. A similar observation was reported by Shakibaie et al. [7], who studied the antifungal activity of selenium nanoparticles against *Aspergillus fumigatus* and *C. albicans,* and found that the MICs for *A. fumigatus* and *C. albicans* were 100 and 70 μg/mL, respectively. However, the high surface-to-volume ratios and their nanoscale sizes provide better activity against biological materials. In addition, Che-SeNPs have significantly lower toxicity than other inorganic and organic forms of supplemental selenium [7].

The current data demonstrated that dietary supplementation with Che-SeNPs substantially affected BW, BWG, feed intake, and feed conversion ratio (FCR). A similar observation was stated by Zhou and Wang [25], who clarified a significant improvement in the FCR and growth performance by supplementation with Che-SeNP up to a 0.5-mg/kg basal diet. Khazraie and Ghazanfarpoor [26] illustrated that weight gain was significantly increased in quail chicks fed the Che-SeNPs-supplemented diet compared to the control. Selim et al. [27], using the Che-SeNPs form (0.15 to 0.30 ppm), showed a marked improvement in BW, BWG, and FCR of broiler chicks. Ibrahim et al. [28] indicated that dietary Che-SeNPs supplementation significantly improved BW, BWG, and FCR of broiler chicks compared to the control group. The improved performance may be due to (1) higher utilization of Che-SeNPs associated with the unique properties of the nano form, such as excellent bioavailability, higher solubility, high cellular uptake, and greater surface activity [2]; (2) the involvement of Se in regulating several enzymatic systems, which interfere in energy metabolism and metabolism of the essential fatty acid apurinic and apyrimidinic base; and (3) Che-SeNPs having high biological activity, immune regulation, and oxidation resistance [22]. In addition, the improved FCR can be elucidated by the Che-SeNPs role in enhancing the activity of intestinal microbiota to digest and absorb the nutrients via the intestinal barriers [9].

The results of the present study in carcass traits and relative organ weight of growing Japanese quails were in line with the study of Khazraie and Ghazanfarpoor [26], who stated that the supplementation of Che-SeNPs to the diet did not affect carcass traits of chicks. Additionally, Cai et al. [6] reported no significant effect of Che-SeNPs on the weights of carcass parts in broilers. Selim et al. [27] indicated that giblets were not affected due to the inclusion of Che-SeNPs in the diet. Recently, Bakhshalinejad et al. [29] reported that neither carcass yield nor carcass yield parts such as thigh and breast muscles and liver, gizzard, and heart of broilers were affected by different Che-SeNPs levels at 42 d of age. In the present study, the relative liver weight was significantly increased with Che-SeNPs; this increase (21–28% relative to control) may be due to the increase in live body weight in Che-SeNPs groups. However, the widespread use of Nano Se in medication and nanoelectronics has increased the risk of their environmental contaminations, which might affect animal species and humans, although it is useful to understand the assessment of the toxicity of Se-NPs to the biological ecosystem. It should be mentioned that the increase in WBCs was insignificant in the Che-SeNPs supplemented-groups; these change along with the change in liver percentage, even if not significant, warrant further investigation to confirm the safety of Che-SeNPs in animal and human nutrition.

Boostani et al. [30] exhibited that packed cell volume, RBCs and WBCs were not different between the birds supplemented with Che-SeNPs and the control birds, which is in line with the current results. Likewise, Chen et al. [31] revealed no significant difference in WBCs, RBCs, and packed cell volume of broilers fed different Se sources. Additionally, Mohamed et al. [32] illustrated that using Che-SeNPs in the diet of Sinai chicks did not significantly affect WBCs, eosinophils, and monocytes. However, our study indicated that Hb level was increased by adding Che-SeNPs, in agreement with Khazraie and Ghazanfarpoor [26], who reported a significant increase in Hb concentration in quails fed a diet containing Che-SeNPs. These findings may be caused by Se enhancing the activity of hemopoietic organs [33]. Se protects the neutrophils, RBCs, WBCs, and other blood components against peroxidative damage [34]. Deficiency of Se can increase ROS in body tissues, the significant adverse impacts on the consistency of immunity cells’ performance and biological membranes [35].

The results of the current study on the blood biochemistry of quails were in agreement with previous studies. Serum total protein and albumin were not significantly affected due to Che-SeNPs supplementation to the broiler diet [27]. However, serum globulin levels were increased with the addition of Che-SeNPs in the diet [36]. Additionally, no significant difference in serum AST activity was observed of chicks fed a diet supplemented with Che-SeNPs [27]. However, our results are similar to the study of Elsaid [37], who reported increased serum ALT activity in birds fed a diet supplemented with Che-SeNPs. Selim et al. [27] found that increasing the Che-SeNPs level in broiler diets increased plasma creatinine levels compared to the control group. However, some studies showed that blood creatinine levels declined in birds fed a diet containing Che-SeNPs Elsaid [37]. The potential reason for these differences is possibly related to the dose and time of animal exposure. We conclude from the current study that the higher Che-SeNPs level is the cause of increased ALT and creatinine as indicators of liver and kidney oxidative damage, whereas lower levels showed less damage.

Selenium has a hypocholesterolemic activity. A significant reduction in plasma TC and an increase in HDL were detected in the Che-SeNPs-treated birds. The dietary addition of nano forms of selenium for hens caused substantial declines in serum levels of cholesterol as compared to that of the control [38]. Rizk [39] stated that Che-SeNPs addition in the chicken diet decreased cholesterol, triglycerides, and low-density lipoproteins and increased HDL compared with the control group. These results might be attributed to the lipolysis that increased with Se intake. Additionally, the reduction of cholesterol may be due to the role of Se in the activation of peroxisome proliferator-activated receptor-γ that can decrease sterol regulatory element-binding protein-2 level, resulting in decreased cholesterol synthesis [40].

The nutritional status of an animal greatly influences the antioxidant system. Se nanoparticles have vital roles in protecting the body cells from reactive oxygen species abundance by decreasing the production of free radicals and lipid peroxidation [41]. Se is well-known for its ability to boost the antioxidant capacity as it forms selenocysteine, a portion of the active center of GSH-peroxidase (Px) [42]. Therefore, a dietary supplementation of Se is essential to improve Se-dependent antioxidant enzymes. These enzymes can help in decreasing the concentration of lipid peroxides and hydrogen peroxide. Dietary Che-SeNPs enhanced oxidative stability and antioxidant ability in broilers [6]. Mohamed et al. [32] reported a positive effect on birds’ plasma total antioxidant capacity when fed a diet containing Che-SeNPs. Aparna and Karunakaran [43] detected an increase in glutathione peroxidase and SOD cellular activity in birds fed Che-SeNPs compared to the control group. El-Deep et al. [4] displayed that Che-SeNPs enhanced the activities of SOD and GSH-Px and reduced MDA content in the liver of broilers. The improvement of antioxidant status in quails fed Che-SeNPs in the current study may be attributed to the fact that (1) Che-SeNPs had high antioxidant activity, because it has an augmented ability to trap free radicals with better antioxidant influence, (2) Che-SeNPs can act as a chemopreventive agent when administered at a smaller particle size, (3) Se plays a vital role as an antioxidant that could protect intestinal mucosa against pathogens and oxidative damage, and (4) Se has immunomodulation properties [44].

Nanominerals such as Che-SeNPs can increase immune parameters and disease resistance [4]. In the current study, we presented a potential approach to the application of Che-SeNPs to improve the immunity of quails. These findings can be due to the higher absorption of selenium nanoparticles. The present data are in harmony with the study of Cai et al. [6], who stated that dietary Che-SeNPs supplementation improved humoral immunity by increasing the levels of IgG and IgM of broiler chicks. Dietary Che-SeNPs supplementation showed immunostimulatory impacts in broiler chicks [45]. The improvement in serum immunoglobulins levels may be attributed to the essential biological role of Che-SeNPs in increasing T helper cells and enhancing the secretion of cytokines [46].

Additionally, Se plays a crucial role in the production of GSH-Px. Selenium inhibits arachidonic acid peroxidation and protects cells and tissues of the immune system from damage caused by free radicals. Therefore, it can be stated that Che-SeNPs boosts birds’ immunity and antioxidant metabolites [45]. Studies have shown that the use of nanominerals in poultry production and its effect on performance and immunity, and reproduction is promising [47,48]. It has been suggested that the application of Se can help to strengthen immunity and decrease inflammation [49,50]. Se, according to Rooke et al. [51], may be involved in a variety of immune functions at the cellular and molecular levels, including lowering immunosuppressive markers such as glucocorticoids; reducing the duration and rate of intramammary infections; and regulating the function of lymphocytes, neutrophils, and natural killer cells. Our results suggest that feeding a diet enriched with Che-SeNPs might have immunostimulatory impacts on quails.

The regulation of microbiota in the gut can be achieved through dietary supplements that can encourage the growth of beneficial bacteria or selectively suppress pathogenic bacteria. Trace elements and natural agents as feed additives may affect the diversity of gut microbiota [8]. The present study found that supplementation of Che-SeNPs in quail diets declined harmful bacteria and increased beneficial bacteria. Se is one of the critical elements that can help microbiota complete its action within the gut [9]. Furthermore, Se supplementation augmented the population of caecum such as *Bifidobacterium* spp. and *Lactobacillus* spp. compared to the basal diet [9]. Therefore, using Che-SeNPs is one of the recommendations for reducing the population of harmful gut bacteria due to its inhibitory effect against many pathogenic bacteria.

Nanotechnology has been found to have advantageous uses in the food chain of humans, mainly through enhancing the bioavailability and delivering enough levels of vital nutrients, vitamins, and minerals in animal products used by humans [10,52,53,54,55,56]. Moreover, the consumers’ demand for foods and their knowledge has been enhanced as consumers want safe and high-quality foods with high sensory quality, favorable health qualities, and prolonged shelf life [57]. Several studies proved the possibility of supplementing nanomaterials to improve mineral contents in animal products; nevertheless, most of these studies were carried out on chicken, meat, and eggs [58,59]. Therefore, more research is needed to analyze the ability of nanomaterials to affect the quality and nutritional content of meat and egg. In addition, the influence of nanomaterials on the environment and health needs further examination [60,61]. Thus, the application of nanoparticles in the poultry industry must be further investigated before they can be applied.

## 5. Conclusions

The current study’s findings demonstrated that dietary supplementation with Che-SeNPs could improve the performance of growing quails. The highest values of growth performance were recorded in the group fed 0.4 g Che-SeNPs g/kg feed during the fattening periods (1–5 wk of age). Moreover, the dietary addition of Che-SeNPs improved the lipid profile, antioxidant indices, and immunity and decreased the intestinal pathogens of growing quails. The groups fed diets supplemented with Che-SeNPs showed lower total yeast and mold count, *Coliform*, *Escherichia coli*, *Enterococcus* spp., and *Salmonella* spp. colonization, and higher lactic acid bacteria counts than those in the control group. However, further studies are warranted to understand the effect of nanominerals and their mechanisms of action, sites of absorption, and transcript expression analysis of distribution.

## Figures and Tables

**Table 1:** Ingredients and nutrient contents of basal diet for growing Japanese quail.

Items	(g/kg)
Ingredient	
Maize 8.5%	518.0
Soybean meal 44%	367.0
Maize gluten meal 62%	52.1
Soybean oil	29.0
Limestone	7.0
Di-calcium phosphate	16.5
Salt	3.0
Premix ^1^	3.0
L-Lysine	1.3
Dl-Methionine	1.1
Choline chloride	2.0
Total	1000
Calculated composition	
Metabolizable energy (MJ/kg)	12.53
Crude protein (g/kg)	240.0
Calcium (g/kg)	8.0
Nonphytate phosphorus (g/kg)	4.5
Lysine (g/kg)	13.0
Total sulphur amino acids (g/kg)	9.2

^1^ Provides per kg of diet: Vitamin A, 12,000 I.U.; Vitamin D3, 5000 I.U.; Vitamin E, 130.0 mg; Vitamin K3, 3.605 mg; Vitamin B1 (thiamin), 3.0 mg; Vitamin B2 (riboflavin), 8.0 mg; Vitamin B6, 4.950 mg; Vitamin B12, 17.0 mg; Niacin, 60.0 mg; D-Biotin, 200.0 mg; Calcium D-pantothenate, 18.333 mg; Folic acid, 2.083 mg; manganese, 100 mg; iron, 80 mg; zinc, 80 mg; copper, 8 mg; iodine, 2 mg; cobalt, 500 mg; and selenium, 150 mg.

**Table 2:** Zone of inhibition produced by Sodium Selenite and selenium nanoparticles.

Item	Sod. Selenite (50 µg/mL)	Selenium Nanoparticles (µg/mL)					DI Water
		50	100	200	400	800	
Bacteria	Inhibition zones (mm)						
*Listeria monocytogenes* ATCC 15313	14 ± 0.2 ^f^	15 ± 0.3 ^e^	19 ± 0.1 ^d^	23 ± 0.2 ^c^	26 ± 0.2 ^b^	32 ± 0.1 ^a^	-
*Staphylococcus aureus* MTTC 1809	11 ± 0.4 ^f^	13 ± 0.2 ^e^	18 ± 0.2 ^d^	20 ± 0.1 ^c^	23 ± 0.3 ^b^	28 ± 0.35 ^a^	-
*Bacillus cereus* ATCC 11778	13 ± 0.2 ^f^	16 ± 0.15 ^e^	20 ± 0.1 ^d^	23 ± 0.2 ^c^	27 ± 0.15 ^b^	33 ± 0.14 ^a^	-
*Escherichia coli* ATCC 25922	9 ± 0.5 ^f^	11 ± 0.45 ^e^	15 ± 0.3 ^d^	17 ± 0.4 ^c^	21 ± 0.2 ^b^	25 ± 0.2 ^a^	-
*Pseudomonas aeruginosa* ATCC 27853	10 ± 0.5 ^f^	11 ± 0.45 ^e^	16 ± 0.4 ^d^	20 ± 0.1 ^c^	22 ± 0.3 ^b^	27 ± 0.19 ^a^	-
*Salmonella enterica* MTCC 1253	8 ± 0.5 ^f^	11 ± 0.45 ^f^	14 ± 0.5 ^d^	17 ± 0.4 ^c^	20 ± 0.5 ^b^	24 ± 0.3 ^a^	-
Fungi							
*Candida albicans* ATCC 4862	11 ± 0.4 ^e^	12 ± 0.3 ^d^	14 ± 0.2 ^c^	15 ± 0.2 ^b^	16 ± 0.15 ^ab^	17 ± 0.1 ^a^	-
*Candida glabrata* ATCC 64677	8 ± 0.5 ^e^	9 ± 0.5 ^d^	9 ± 0.5 ^d^	10 ± 0.4 ^c^	11 ± 0.4 ^b^	13 ± 0.2 ^a^	-
*Candida parapsilosis* ATCC 22019	10 ± 0.35 ^d^	11 ± 0.4 ^c^	12 ± 0.3 ^b^	13 ± 0.3 ^b^	14 ± 0.2 ^a^	14 ± 0.3 ^a^	-
*Candida guilliermondii* ATCC 6260	8 ± 0.5 ^d^	8 ± 0.5 ^d^	9 ± 0.5 ^c^	9 ± 0.5 ^c^	10 ± 0.3 ^b^	11 ± 0.5 ^a^	-

Mean ± SE, Means in the same row with a similar superscript letter following them are not significantly different (*p* < 0.05).

**Table 3:** The MIC (Minimum Inhibitory Concentration), MBC (Minimum Bactericidal Concentration), and MFC (Minimum fungicidal concentration) of the selenium nanoparticles.

Microorganisms	Selenium Nanoparticles	
	MIC µg/mL	MBC µg/mL
Bacteria		
*Listeria monocytogenes* ATCC 15313	30	60
*Staphylococcus aureus* MTTC 1809	35	70
*Bacillus cereus* ATCC 11778	25	50
*Escherichia coli* ATCC 25922	45	90
*Pseudomonas aeruginosa* ATCC 27853	40	80
*Salmonella enterica* MTCC 1253	50	100
Fungi		
*Candida albicans* ATCC 4862	70	140
*Candida glabrata* ATCC 64677	90	180
*Candida parapsilosis* ATCC 22019	80	160
*Candida guilliermondii* ATCC 6260	100	200

**Table 4:** Growth performance of growing Japanese quail as affected by dietary treatments.

Items	Chemical Nano Selenium Levels (g/kg Diet)				SEM	*p* Value	
	0	0.2	0.4	0.6		Linear	Quadratic
Body weight (g)							
1 wk	27.1	27.2	27.2	27.2	0.049	0.812	0.598
3 wk	92.6 ^b^	98.4 ^a^	97.5 ^a^	97.8 ^a^	0.717	0.002	0.006
5 wk	178.5^c^	190.8 ^b^	198.1 ^a^	193.7 ^ab^	1.354	<0.0001	0.0004
Body weight gain (g/day)							
1–3 wk	4.67 ^b^	5.09 ^a^	5.02 ^a^	5.04 ^a^	0.051	0.003	0.006
3–5 wk	6.14 ^c^	6.60 ^b^	7.18 ^a^	6.85 ^ab^	0.101	0.001	0.008
1–5 wk	5.41 ^c^	5.84 ^b^	6.10 ^a^	5.95 ^ab^	0.051	<0.0001	0.0005
Feed intake (g/day)							
1–3 wk	14.1 ^a^	12.9 ^c^	13.2 ^b^	13.1 ^bc^	0.079	0.0002	0.0002
3–5 wk	23.7 ^a^	19.9 ^c^	21.0 ^b^	20.2 ^c^	0.202	<0.0001	0.0001
1–5 wk	18.9 ^a^	16.4 ^c^	17.1 ^b^	16.7 ^c^	0.083	<0.0001	<0.0001
Feed conversion ratio (g/g)							
1–3 wk	3.01 ^a^	2.53 ^b^	2.63 ^b^	2.60 ^b^	0.038	0.0002	0.0005
3–5 wk	3.86 ^a^	3.02 ^b^	2.93 ^c^	2.95 ^bc^	0.018	<0.0001	<0.0001
1–5 wk	3.49 ^a^	2.81 ^b^	2.81 ^b^	2.80 ^b^	0.015	<0.0001	<0.0001

Means in the same row with no superscript letters after them or a similar superscript letter following them are not significantly different (*p* < 0.05).

**Table 5:** Carcass traits and relative organs of growing Japanese quail as affected by dietary treatments.

Items	Chemical Nano Selenium Levels (g/kg Diet)				SEM	*p* Value	
	0	0.2	0.4	0.6		Linear	Quadratic
Carcass %	73.7	76.1	71.7	72.8	1.087	0.199	0.566
Liver %	2.22 ^b^	2.84 ^a^	2.85 ^a^	2.69 ^a^	0.099	0.036	0.014
Gizzard %	2.54	2.30	2.10	2.35	0.208	0.531	0.372
Heart %	1.01	1.14	1.03	0.94	0.076	0.473	0.267
Giblets %	5.76	6.28	5.98	5.98	0.344	0.838	0.506
Dressing %	79.4	82.4	77.7	78.8	1.279	0.300	0.509

Means in the same row with no superscript letters after them or a similar superscript letter following them are not significantly different (*p* < 0.05).

**Table 6:** Hematological parameters of growing Japanese quail as affected by dietary treatments.

Items ^1^	Chemical Nano Selenium Levels (g/kg Diet)				SEM	*p* Value	
	0	0.2	0.4	0.6		Linear	Quadratic
WBCs (10^3^/μL)	22.4	23.3	23.7	23.7	1.972	0.575	0.741
LYM (%)	93.7	93.2	93.1	94.6	0.629	0.349	0.163
MID (%)	5.84	6.41	6.50	3.14	0.771	0.059	0.042
GRA (%)	0.32	0.44	0.36	0.17	0.084	0.269	0.175
RBCs (10^6^/μL)	2.34	2.60	2.63	2.75	0.196	0.194	0.749
HGB (g/dL)	9.27 ^b^	11.8 ^ab^	12.4 ^a^	13.4 ^a^	0.822	0.001	0.399
HCT (%)	32.3	35.8	29.4	21.2	3.438	0.032	0.127
MCV (µm^3^)	137.8	137.9	127.1	124.9	2.985	0.014	0.758
MCH (pg)	40.9 ^c^	47.9 ^b^	53.9 ^a^	56.9 ^a^	1.167	<0.0001	0.163
RDWSD	51.9 ^a^	52.4 ^a^	44.0 ^ab^	41.5 ^b^	2.420	0.009	0.574
RDWCV	13.1 ^a^	13.3 ^a^	11.9 ^b^	11.2 ^b^	0.349	0.002	0.222
PLT (10^3^/μL)	5.67 ^b^	8.67 ^b^	16.7 ^a^	15.0 ^a^	2.771	0.032	0.476

Means in the same row with no superscript letters after them or with a common superscript letter following them are not significantly different (*p* < 0.05). ^1^ WBCs: white blood cells; LYM: lymphocytes; MID: mid-range; GRA: granulocytes; RBCs: red blood cells; HGB: hemoglobin; HCT: hematocrit; MCV: Mean corpuscular volume; MCH: Mean corpuscular hemoglobin; RDWSD: Red blood cell distribution width; RDWCV: Red blood cell distribution volume; PLT: Platelet count.

**Table 7:** Liver and kidney function of growing Japanese quail as affected by dietary treatments.

Items ^1^	Chemical Nano Selenium Levels (g/kg Diet)				SEM	*p* Value	
	0	0.2	0.4	0.6		Linear	Quadratic
TP (g/dL)	3.27	2.88	3.22	3.35	0.109	0.332	0.067
ALB (g/dL)	1.20	1.21	1.27	1.16	0.026	0.707	0.086
GLOB (g/dL)	1.63 ^c^	1.72 ^c^	1.95 ^b^	2.19 ^a^	0.066	0.0003	0.329
A/G (%)	0.74 ^a^	0.71 ^a^	0.65 ^a^	0.53 ^b^	0.022	0.0005	0.122
AST (IU/L)	221.2	229.0	237.8	238.3	4.207	0.031	0.500
ALT (IU/L)	10.8 ^c^	12.9 ^b^	13.7 ^b^	16.67 ^a^	0.493	<0.0001	0.415
LDH (IU/L)	119.5 ^c^	143.5 ^a^	133.2 ^b^	144.0 ^a^	1.328	<0.0001	0.0001
Creatinine (mg/dL)	0.33 ^b^	0.33 ^b^	0.42 ^a^	0.44 ^a^	0.017	0.0008	0.660
Urea (mg/dL)	6.86	7.03	7.12	7.24	0.098	0.050	0.839

Means in the same row with no superscript letters after them or a similar superscript letter following them are not significantly different (*p* < 0.05). ^1^ TP: total protein; ALB: albumin; GLOB: globulin; A/G: albumin/ globulin ratio; LDH: Lactate dehydrogenase, AST: aspartate aminotransferase and ALT: alanine aminotransferase.

**Table 8:** Lipid profile of growing Japanese quail as affected by dietary treatments.

Items ^1^	Chemical Nano Selenium Levels (g/kg Diet)				SEM	*p* Value	
	0	0.2	0.4	0.6		Linear	Quadratic
TC (mg/dL)	153.6 ^a^	143.4 ^b^	144.5 ^ab^	125.3 ^c^	1.912	0.0002	0.159
TG (mg/dL)	298.8 ^a^	225.0 ^b^	210.0 ^bc^	192.0 ^c^	5.882	<0.0001	0.002
HDL (mg/dL)	35.3 ^c^	46.1 ^b^	56.8 ^a^	38.92 ^bc^	3.092	0.164	0.002
LDL (mg/dL)	58.5 ^a^	52.3 ^b^	45.7 ^c^	47.94 ^c^	2.911	0.024	0.204
VLDL (mg/dL)	59.8 ^a^	45.0 ^b^	42.0 ^bc^	38.40 ^c^	1.176	<0.0001	0.002
SOD (U/mL)	0.12 ^c^	0.22 ^b^	0.29 ^a^	0.22 ^b^	0.004	<0.0001	<0.0001
MDA (nmol/mL)	0.33 ^a^	0.24 ^b^	0.22 ^b^	0.13 ^c^	0.007	<0.0001	0.792
GSH (ng/mL)	0.11 ^c^	0.22 ^b^	0.28 ^a^	0.26 ^a^	0.008	<0.0001	0.0001
GPX (mg/dL)	0.13 ^d^	0.23 ^c^	0.30 ^b^	0.34 ^a^	0.007	<0.0001	0.006
IgG (mg/dL)	0.89 ^b^	1.33 ^a^	1.19 ^a^	1.17 ^a^	0.042	0.013	0.002
IgM (mg/dL)	0.49 ^c^	0.56 ^bc^	0.64 ^b^	0.90 ^a^	0.026	<0.0001	0.024
IgA (mg/dL)	0.53 ^b^	0.64 ^b^	0.80	0.85 ^a^	0.034	0.0007	0.547
Selenium	0.07 ^c^	0.19 ^b^	0.24 ^ab^	0.27 ^a^	0.016	0.0001	0.060

Means in the same row with no superscript letters after them or a similar superscript letter following them are not significantly different (*p* < 0.05). ^1^ TC: total cholesterol; TG: triglycerides; HDL: high-density lipoprotein; LDL: low-density lipoprotein. SOD: superoxide dismutase; MDA: malondialdehyde; GSH: reduced glutathione; GPX: glutathione peroxidase; IgG and M: immunoglobulin G.

**Table 9:** Caecal microbiota of growing Japanese quail as affected by dietary treatments.

Items	Chemical Nano Selenium Levels (g/kg Diet)				SEM	*p* Value	
	0	0.2	0.4	0.6		Linear	Quadratic
Microbiological count (Log CFU/g)							
TBC	6.05 ^a^	5.32 ^b^	5.08 ^c^	5.31 ^b^	0.006	<0.0001	<0.0001
TYMC	5.82 ^a^	5.20 ^b^	4.90 ^d^	5.12 ^c^	0.014	<0.0001	<0.0001
*Coliform*	5.96 ^a^	5.26 ^b^	5.08 ^c^	4.92 ^d^	0.035	<0.0001	0.0004
*Escherichia coli*	5.93 ^a^	5.25 ^b^	5.05 ^c^	5.09 ^c^	0.022	<0.0001	<0.0001
Lactic acid bacteria	5.31 ^c^	5.58 ^a^	5.40 ^b^	5.53 ^a^	0.017	0.0003	0.0048
*Enterococcus* spp.	5.86 ^a^	5.23 ^b^	4.97 ^c^	5.08 ^c^	0.026	<0.0001	<0.0001
*Salmonella* spp.	6.38 ^a^	3.22 ^b^	2.23 ^c^	2.07 ^c^	0.057	<0.0001	<0.0001

Means in the same row with no superscript letters after them or a similar superscript letter following them are not significantly different (*p* < 0.05). TBC: Total bacterial count. TYMC: total yeasts and molds count.

## Data Availability

All data are published in the cited literature and reported in the text of this manuscript.
